# Conformational Properties of Seven Toac-Labeled Angiotensin I Analogues Correlate with Their Muscle Contraction Activity and Their Ability to Act as ACE Substrates

**DOI:** 10.1371/journal.pone.0136608

**Published:** 2015-08-28

**Authors:** Luis Gustavo D. Teixeira, Luciana Malavolta, Patrícia A. Bersanetti, Shirley Schreier, Adriana K. Carmona, Clovis R. Nakaie

**Affiliations:** 1 Department of Biophysics, Escola Paulista de Medicina, Universidade Federal de Sao Paulo, Sao Paulo, Sao Paulo, Brazil; 2 Department of Physiological Sciences, Santa Casa de Sao Paulo School of Medical Sciences, Sao Paulo, Sao Paulo, Brazil; 3 Department of Health and Informatics, Universidade Federal de São Paulo, Sao Paulo, Sao Paulo, Brazil; 4 Department of Biochemistry, Institute of Chemistry, Universidade de Sao Paulo, Sao Paulo, Sao Paulo, Brazil; University of São Paulo, BRAZIL

## Abstract

Conformational properties of the angiotensin II precursor, angiotensin I (AngI) and analogues containing the paramagnetic amino acid TOAC (2,2,6,6-tetramethylpiperidine-1-oxyl-4-amino-4-carboxylic acid) at positions 0, 1, 3, 5, 8, 9, and 10, were examined by EPR, CD, and fluorescence. The conformational data were correlated to their activity in muscle contraction experiments and to their properties as substrates of the angiotensin I-converting enzyme (ACE). Biological activity studies indicated that TOAC^0^-AngI and TOAC^1^-AngI maintained partial potency in guinea pig ileum and rat uterus. Kinetic parameters revealed that only derivatives labeled closer to the N-terminus (positions 0, 1, 3, and 5) were hydrolyzed by ACE, indicating that peptides bearing the TOAC moiety far from the ACE cleavage site (Phe^8^-His^9^ peptide bond) were susceptible to hydrolysis, albeit less effectively than the parent compound. CD spectra indicated that AngI exhibited a flexible structure resulting from equilibrium between different conformers. While the conformation of N-terminally-labeled derivatives was similar to that of the native peptide, a greater propensity to acquire folded structures was observed for internally-labeled, as well as C-terminally labeled, analogues. These structures were stabilized in secondary structure-inducing agent, TFE. Different analogues gave rise to different β-turns. EPR spectra in aqueous solution also distinguished between N-terminally, internally-, and C-terminally labeled peptides, yielding narrower lines, indicative of greater mobility for the former. Interestingly, the spectra of peptides labeled at, or close, to the C-terminus, showed that the motion in this part of the peptides was intermediate between that of N-terminally and internally-labeled peptides, in agreement with the suggestion of turn formation provided by the CD spectra. Quenching of the Tyr^4^ fluorescence by the differently positioned TOAC residues corroborated the data obtained by the other spectroscopic techniques. Lastly, we demonstrated the feasibility of monitoring the progress of ACE-catalyzed hydrolysis of TOAC-labeled peptides by following time-dependent changes in their EPR spectra.

## Introduction

The renin–angiotensin system (RAS) exerts an important role in cardiovascular and hydro-electrolyte homeostasis [1–4]. Several approaches to the treatment of diseases related to these processes involve drugs that will act on the RAS. In addition, recent studies demonstrated that newly discovered components of the RAS are related to other pathologies, such as cancer, inflammation, and glaucoma [4]. One longtime known event of the RAS cascade is the conversion of the decapeptide angiotensin I (AngI, DRVYIHPFHL) to the octapeptide angiotensin II (AngII, DRVYIHPF), the ligand of GPCRs that trigger signal transduction leading to increase of blood pressure [1–4].

Cleavage of the C-terminal dipeptide is catalyzed by the metallopeptidase angiotensin I-converting enzyme (EC 3.4.15.1, ACE) [5]. In view of the physiological and pathological importance of this process, a large amount of work has been devoted to the detailed understanding of the elements of this reaction. Moreover, knowledge about conformation and dynamics of the reaction substrate and product should enable the design of more efficient drugs for the treatment of RAS-related diseases.

Interestingly, while an extensive literature has been produced concerning AngII and its active (agonists or antagonists) or inactive analogues, a much smaller amount of data exists focusing on AngI [6–15]. More recent ^1^H NMR studies in DMSO or H_2_O/TFE 34%/66% v/v evinced the existence of a turn in the C-terminal portion of AngI, which is stabilized in the former solvent [12–13]. Molecular dynamics simulations corroborated this result.

Studies of the conformational properties of peptides have made use, among other spectroscopic techniques, of electron paramagnetic resonance (EPR) spectroscopy by means of incorporation of the non-coded paramagnetic amino acid TOAC (2,2,6,6-tetramethylpiperidine-1-oxyl-4-amino-4-carboxylic acid) whose introduction allowed linking a spin label to the peptide backbone via a peptide bond [16]. Initially, TOAC was incorporated at the peptide’s N-terminus [16–17]; subsequently a new synthetic procedure rendered viable its incorporation in any position of the sequence [18].

As a result, a large amount of applications have been reported in the literature [19], expanding the potentiality of EPR spectroscopy to provide information about peptide conformation and dynamics. This is basically due to the fact that the EPR spectra of this cyclic amino acid spin label are highly sensitive to the motion and orientation of coupled (macro) molecules due to the fact that, in addition to its linking via a peptide bond, its constrained C^α,α^-tetrasubstituted cyclic structure hampers rotation about side chain bonds, leading to formation of bends in the peptide backbone [20]. Moreover, since TOAC, similarly to aminoisobutyric acid (Aib), is a disubstituted glycine, it favors acquisition of α- and 3_10_-helical conformations [20–21].

Structure-function studies of TOAC-carrying peptides have been extensively performed by our group, AngII being the first biologically active peptide investigated [16–18]. Conformational studies in solution and in the presence of model membranes, making use of additional techniques such as circular dicroism (CD) and fluorescence, of AngII and another vasoactive peptide, bradykinin (BK), as well as their TOAC-labeled derivatives, have been reported [22–23]. The effect of TOAC’s introduction on biological activity of these peptides was also investigated [24–25]. Labeling of other signaling peptides [26–28] showed that labeled α-MSH displayed full biological activity.

A novel approach was undertaken by examining the interaction between the parent hormones AngII and BK, as well as their active and inactive TOAC analogues, and constructs of their respective GPCRs, AT_1_R and BKRB_1_, containing binding sites for the hormones [29–30]. In another approach to broaden the use of TOAC-containing peptides, we presented a preliminary report describing the possibility of studying the catalytic activity of ACE upon AngI bearing TOAC at positions 1, 3, 8 or 9 [31]. The enzyme hydrolyzes the scissile bond Phe^8^-His^9^ of AngI, releasing the vasoconstrictor AngII [5]. Although AngI and AngII differ by only two amino acid residues, the different line shapes of their EPR spectra allowed monitoring the enzyme-catalyzed conversion.

In the present work we describe the synthesis of additional AngI analogues carrying TOAC at positions 0, 5, or 10. To our knowledge this is the first time a peptide is labeled at the C-terminus. Thus, an almost complete TOAC-scan was performed, where a total of seven TOAC-carrying analogs were evaluated from the point of view of their conformational properties using EPR, CD, and fluorescence spectroscopy. In order to look for structure-activity relationships the pharmacological activity of the peptides was examined in muscle contraction experiments (guinea pig ileum and rat uterus), according to ref. [31], and their susceptibility to hydrolysis by ACE was evaluated both by analysis of reactants and products, and by time-dependent changes in the EPR spectra.

## Materials and Methods

### Materials

*Tert*-butyloxycarbonyl (Boc) and 9-fluorenylmethyloxycarbonyl (Fmoc) amino acids were purchased from Bachem (Torrance, CA, USA) and purified rabbit lung ACE was from Sigma (St. Louis, MO, USA). Dimethylformamide (DMF) and ninhydrin were distilled over P_2_O_5_ and under reduced pressure, respectively. All solvents were HPLC grade and all chemicals met ACS standards. The molar concentration of rabbit lung ACE was determined by active site titration with lisinopril, as previously described [32–33].

### Methods

#### Peptide Synthesis

TOAC-containing AngI derivatives were synthesized manually according to the combined Boc/Fmoc strategies as previously reported [18, 31]. All synthetic steps were performed through Fmoc chemistry [34] and anhydrous HF (Boc chemistry) [35] was used for removal of the peptide from the solid support. The C-terminally coupled TOAC^10^-AngI peptide was synthesized starting from TOAC-copoly(styrene-1% divinylbenzene) support previously obtained by attaching Fmoc-TOAC to a chloromethyl-resin according to a standard protocol [35]. The crude spin-labeled peptides were submitted to alkaline treatment (pH 10, for 1 h at 50°C) for complete reversal of the N-O protonation that occurs during the HF cleavage reaction.

Unlabeled peptides were synthesized using the Boc strategy. The peptides were purified by preparative HPLC (C_18_-column) using aqueous 0.02 M ammonium acetate (pH 5) and 60% acetonitrile solutions as solvents A and B, respectively (linear gradient of 30–70% B for 2 h, flow rate of 10 mL/min). Peptide homogeneity was determined through analytical HPLC, mass spectrometry and amino acid analysis. All peptides were obtained in satisfactory yield; the synthesis scale was 0.2 mmol.

#### Analytical RP-HPLC

RP-HPLC analyses were carried out in a TFA/acetonitrile gradient using a Waters Associates HPLC system consisting of two 510 HPLC pumps, an automated gradient controller, a Rheodyne manual injector, a 486 UV detector, and a 746 data module. The column employed was a Vydac C18 column (0.46 x 15 cm, 5 μm particle size, 300 Å pore size), detection at λ = 210 nm, using the solvent systems A: 0.1% TFA/H_2_O and B: 60% acetonitrile/0.1% TFA/H_2_O. A linear gradient of 5–95% B was used (30 min, flow rate 1.5 mL/min).

#### Mass Spectrometry

The liquid chromatography/electrospray ionization mass spectrometry (LC/ESI-MS) experiments were performed on a system consisting of a Waters Alliance model 2690 separations module and model 996 photodiode array detector (Waters, Eschborn, Germany) controlled with a Compaq AP200 workstation coupled to a Micromass model ZMD mass detector (Micromass, Altrincham, UK). The samples were automatically injected on a Waters narrow bore Nova-Pak column C_18_ (2.1 × 150 mm, 60 Å pore size, 3.5 μm particle size). The elution was carried out with solvents A (0.1% TFA/H_2_O) and B (60% acetonitrile/0.1% TFA/H_2_O) at a flow rate of 0.4 mL/min using a linear gradient from 5% to 95% B in 30 min. The condition used for mass spectrometry measurements was a positive ESI. Specifically for the case of monitoring enzymatic reactions, a different linear gradient of 35 to 50% (v/v) of solution B in 15 min, with flow rate of 1.5mL/min was used.

#### Amino acid analysis

Peptide composition was monitored using amino acid analysis performed on a Biochrom 20 Plus amino acid analyzer (Pharmacia LKB Biochrom Ltd., Cambridge, UK) equipped with an analytical cation-exchange column.

#### EPR studies

Spectra were obtained at 9.5 GHz in a Bruker ER 200 spectrometer at room temperature (22 ± 2°C) using flat quartz cells from Wilmad Glass Co., Buena, NJ, USA. The magnetic field was modulated with amplitudes less than one-fifth of the line widths, and the microwave power was 5 mW to avoid saturation effects. Rotational correlation times (τ_B_ and τ_C_) of the paramagnetic compounds were calculated from spectral line heights and line widths according to Schreier *et al*. [36]. Only the latter parameter will be reported here. The concentration of peptides was 100 μM in 0.02 M phosphate buffer, pH 7.0. Equivalent results were obtained in triplicate measurements.

#### CD studies

CD spectra were obtained on a Jasco J-810 spectropolarimeter at room temperature continually flushed with ultra-pure nitrogen. Peptide concentration was: 100 μM in 0.02 M phosphate buffer, pH 7.0 or with addition of TFE (up to 90%, v/v). Equivalent results were obtained in triplicate measurements.

#### Fluorescence studies

Static fluorescence spectra were obtained at room temperature (22 ± 2°C) in a Hitachi F 2500 spectrofluorimeter (Hitachi, Tokyo), using cuvettes with excitation path lengths of 2 mm or 5 mm and emission path lengths of 10 mm. Excitation and emission slits were 5 nm and the peptides concentration was 100 μM in 0.02 M phosphate buffer, pH 7.0. The peptides Tyr^4^ residue was excited at 275 nm. Equivalent results were obtained in triplicate measurements.

#### Bioassays

The biological potencies of AngI and their TOAC attaching derivatives were examined in rat uterus and guinea-pig ileum, accordingly to previous report [24]. Briefly, uterine horns from female rats (200–240 g), which received 100 mg diethylstilbestrol per 100g weight 24 h before the experiments, were removed and mounted in 5-mL organ baths containing De Jalon’s solution which was bubbled with a gas mixture of 95% O_2_-5% CO_2_ and the temperature was kept at 30°C in order to inhibit the spontaneous contractions observed at higher temperatures.

For contractile studies in guinea-pig ileum, the lower portion of ileum was excised from guinea-pigs (220–250 g) and washed in Tyrode solution. Segments of 4-cm length were suspended in 5 mL organ baths which contained aerated Tyrode solution bubbled with a gas mixture of 95% O_2_−5% CO_2_ and the temperature was kept at 37 ± 0.5°C.

The concentration-response curves were obtained by administration of successive treatments with the agonist for 90 s, at 5-min intervals. Intrinsic activities of paramagnetic analogues were estimated by the maximum response relative to that of parent peptide (AngI). As early determined [31], the biological potencies of AngI in rat uterus and guinea-pig ileum were 1% and 11%, respectively, in comparison with those of AngII, taken as 100%.

#### Determination of kinetic parameters for ACE activity

The hydrolysis of peptides by purified rabbit lung ACE was performed at 37°C in 0.1 M Tris-HCl buffer containing 50 mM NaCl and 10 μM ZnCl_2_ at pH 7.0 (1.0 mL final volume). The hydrolysis reaction and all released peptide products were monitored by LC/ESI-MS as a function of time as already described [31], and the peak areas were used for determination of kinetic parameters. The enzyme concentration (0.44 nM) was chosen so as to hydrolyze less than 5% of the substrate present in order to obtain the initial reaction rates. The peptide concentrations were 1, 5, 10, 15 and 20 (x 10^−5^ M). The hydrolysis reaction was interrupted at different times by adding 0.1% TFA aqueous solution. The *k*cat, *K*m and *k*cat*/K*m values were obtained by analysis of the non-linear regression data using the GraFit program (Erithacus Software). The standard deviation of these parameters was less than 5%.

## Results and Discussion

We have previously reported studies of ACE’s enzymatic activity upon AngI and its analogues containing TOAC at positions 1, 3, 8, and 9 in conjunction with preliminary EPR and fluorescence data [31]. Here we report studies of muscle contraction activity and of the ability to act as ACE’s substrates, of three new AngI derivatives labeled at positions 0, 5, and 10, as well as more detailed EPR and fluorescence studies of the parent compound and all seven TOAC-labeled analogues. The effect of TFE on the peptides CD spectra was also examined. The biological activity of the spin-labeled peptides is correlated with their conformational properties.

### Muscle contraction activity

The peptides behavior in contraction experiments was determined in guinea pig ileum and rat uterus following standard procedures [24–25, 31]. ACE is known to convert AngI to the strongly vasoactive and spasmogenic peptide AngII, which, in turn, induces contraction of muscle tissues. In agreement with the earlier results for TOAC^1^-AngI [31], the TOAC^0^-AngI analogue also retained partial biological potency, with two-fold and 18% that of AngI in rat uterus and guinea pig ileum, respectively. None of the other TOAC-AngI derivatives (labeled at positions 3, 5, 8, 9 and 10) showed biological potency, in close accordance with previous observations that the N-terminal portion of released AngII is less important for maintaining its potency [37–38]. These results are analogous to those of Bettio *et al*. who found that, with regard to biological activity, introduction of TOAC in the N-terminal part of neuropeptide Y, a GPCR ligand, was better tolerated than in proximity to the C-terminus [39].

### ACE activity studies

ACE’s peptidase activity upon AngI and its TOAC-containing derivatives was examined as described in Methods. The reaction progress was monitored through LC/EMI-MS of the components in solution. The results indicated that AngI derivatives carrying TOAC at positions 0, 1, 3, and 5 were cleaved by ACE, while the remaining analogues were not. While TOAC^8^-AngI and TOAC^9^-AngI lack the ability to act as substrates, possibly due to the fact the TOAC residue occupies either position P_1_ or position P’_1_, respectively, of the AngI P_1_-P’_1_ scissile bond, C-terminally labeled TOAC^10^-AngI does not act as a substrate due to proximity of the spin label to the cleavage site, possibly creating steric hindrance. In this case, both the bulkiness of TOAC and the turn promoted by this residue (see below) would prevent fitting of the sequence to be cleaved to the enzyme’s active site.

The kinetic parameters calculated for AngI and its TOAC derivatives (Table 1) suggest that ACE’s hydrolytic activity depends on the distance between the cleavage site and the inserted TOAC moiety. Accordingly, the k_cat_/K_m_ value for AngI was almost eight times higher than that for the weakest TOAC^5^-AngI substrate (15.4 μM^-1^.min^-1^ *vs* 2 μM^-1^.min^-1^). These results are in accord with previous work [40–41] that revealed that the greater the distance of a modified residue from the cleavage site, the greater the likelihood of the peptide being cleaved by ACE.

**Table 1:** Kinetic parameters for hydrolysis of AngI and its TOAC-analogues by purified rabbit lung ACE^a^.

Peptide	*K*_m_ (μM)	*k*_cat_ (min^-1^)	*k*_cat_/*K*_m_ (μM^-1^.min^-1^)
AngI	30.7	472.7	15.4
TOAC^0^-AngI	33.2	394.7	11.9
TOAC^1^-AngI^b^	38.1	350.0	9.2
TOAC^3^-AngI^b^	47.3	153.7	3.2
TOAC^5^-AngI	75.6	151.3	2.0
TOAC^8^-AngI^b^ ^,^ ^c^	-	-	-
TOAC^9^-AngI^b^ ^,^ ^c^	-	-	-
TOAC^10^-AngI^c^	-	-	-

^a^Experimental conditions: see Methods.

^b^From Teixeira et al., 2007 [31].

^c^Not hydrolyzed by ACE.

When muscle contraction experiments are examined in the light of ACE activity, it is seen that in the case of TOAC^8^-, TOAC^9^-, and TOAC^10^-AngI analogues, when these peptides are exposed to ACE, they fail in being converted to the corresponding spin-labeled AngII. However, this is not the case for TOAC^3^- and TOAC^5^-AngI, since these analogues do act as substrates for the enzyme. These results indicate that although the latter analogues are converted to their AngII counterpart, these products are not accepted as ligands for AngII receptors. Thus, the active site of ACE is less selective than the receptor site where AngII binds to trigger muscle contraction.

When muscle contraction experiments are examined in the light of ACE activity, it is seen that in the case of TOAC^8^-, TOAC^9^-, and TOAC^10^-AngI analogues, when these peptides are exposed to ACE, they fail in being converted to the corresponding spin-labeled AngII. However, this is not the case for TOAC^3^- and TOAC^5^-AngI, since these analogues do act as substrates for the enzyme. These results indicate that although the latter analogues are converted to their AngII counterpart, these products are not accepted as ligands for AngII receptors. Thus, the active site of ACE is less selective than the receptor site where AngII binds to trigger muscle contraction.

### EPR studies

**Fig 1:**
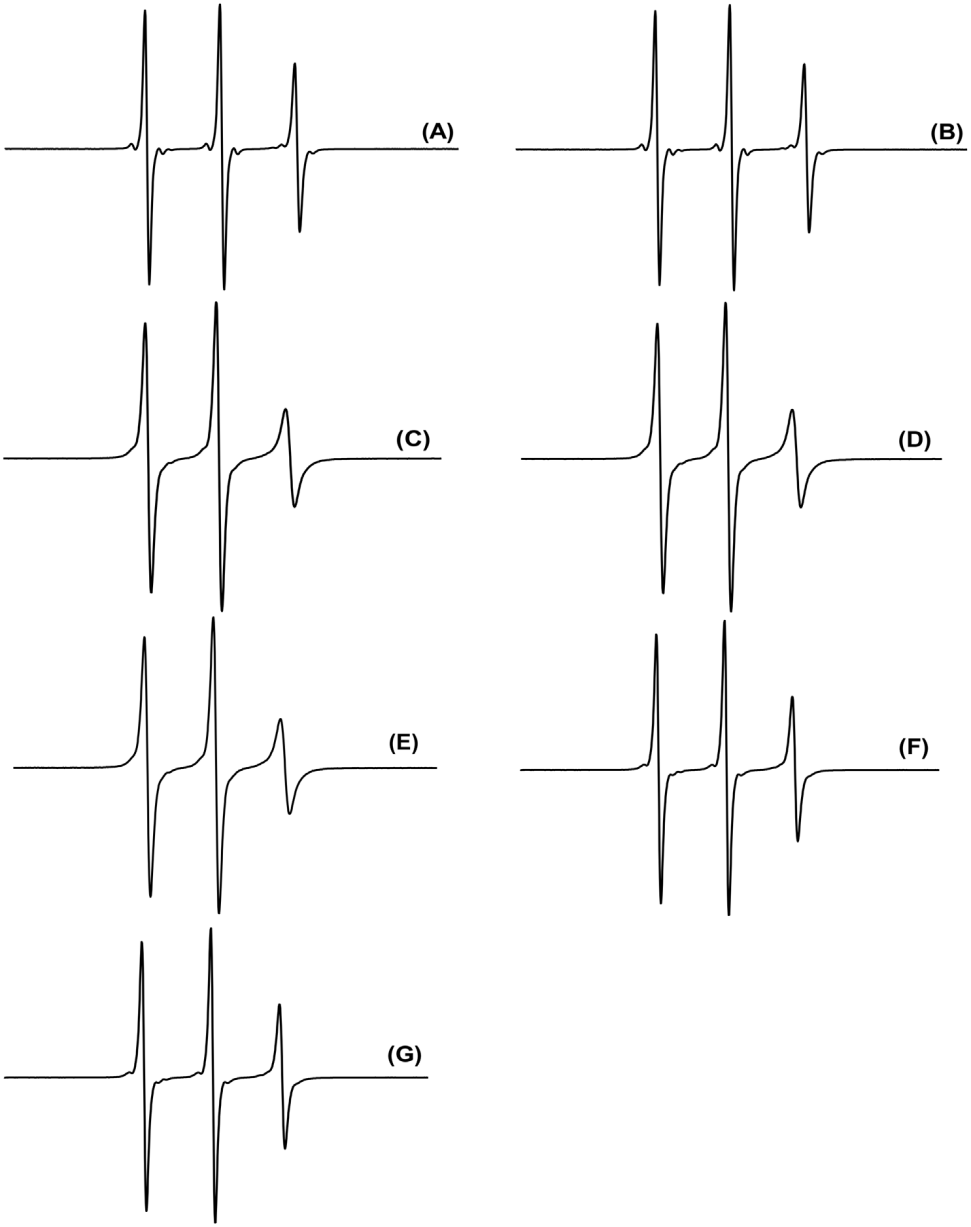
EPR spectra of TOAC^0^-AngI (A), TOAC^1^-AngI (B), TOAC^3^-AngI (C), TOAC^5^-AngI (D), TOAC^8^-AngI (E), TOAC^9^-AngI (F) and TOAC^10^-AngI (G) in 20 mM phosphate buffer, pH 7.0. Field scan: 100 Gauss.

**Fig 2:**
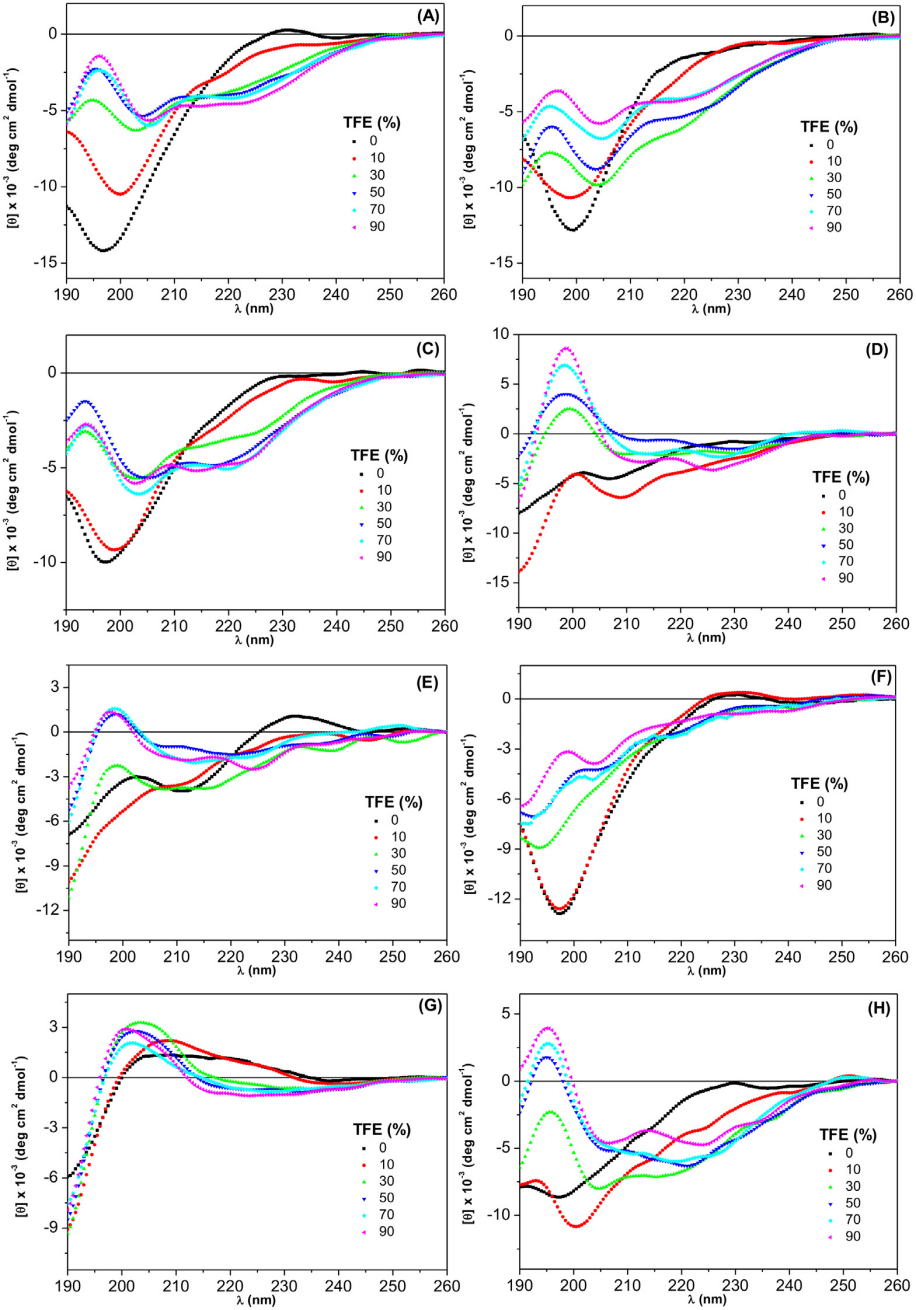
CD spectra of AngI (A), TOAC^0^-AngI (B), TOAC^1^-AngI (C) TOAC^3^-AngI (D), TOAC^5^-AngI (E), TOAC^8^-AngI (F), TOAC^9^-AngI (G) and TOAC^10^-AngI (H) as a function of TFE concentration.

Fig 1 presents the EPR spectra of TOAC-containing AngI analogues in aqueous solution. The three narrow lines are in agreement with a small molecule tumbling freely in solution. Small peptides in aqueous solution are known to be in equilibrium between different conformations. It is noteworthy that the internally TOAC-labeled analogues (positions 3, 5 and 8) gave rise to spectra with broader linewidths, suggesting that the molecular motion at these sites is comparatively more restricted. At least two factors could contribute to this behavior: the very fact that these residues are located internally in the peptide chain and the known ability of TOAC, a cyclic disubstituted glycine, to promote a structure-bending effect [20]. CD spectra presented in the next section (Fig 2) are suggestive of the presence of bends (turns) in C-terminally labeled TOAC^9^-AngI and TOAC^10^-AngI, but not in the spectra of N-terminally labeled TOAC^0^-AngI and TOAC^1^-AngI.

Calculated rotational correlational times (τ_C_) (Table 2, [36]) for spectra in Fig 1 show that τ_C_ values vary in the order TOAC^1^-AngI < TOAC^0^-AngI < TOAC^9^-AngI < TOAC^10^-AngI < TOAC^8^AngI < TOAC^3^-AngI < TOAC^5^-AngI. Table 2 also presents the isotropic hyperfine splitting (a_0_) measured from the spectra of TOAC-labeled analogs. A slight difference is seen between the values for TOAC at the peptides N-terminus and in the other analogues. Although very small, this difference is in line with the more restricted motion in the backbone and in the C-terminal analogues and could be related to the greater flexibility of the N-terminal region, that, on the average, would expose more this region to the bulk aqueous phase, therefore, to a slightly higher polarity.

**Table 2:** Values of rotational correlation times (τ_C_) of AngI and AngII TOAC analogues calculated from the peptides EPR spectra, values of isotropic hyperfine splittings (a_0_) measured in the EPR spectra of AngI TOAC analogues, and percentage of quenching of TOAC-AngI analogues fluorescence.

TOAC position		AngI		AngII
	τ_C_ x 10^−10^, s^a^	a_0_ (Gauss)^b^	% fluorescence quenching	τ_C_ x 10^−10^, s^c^
0	2.7	16.6	42.5	2.6
1	2.8	16.5	63.4	2.7
3	7.6	16.2	75.6	6.4
5	8.7	16.2	71.2	7.2
8	7.4	16.2	26.2	n.d
9	3.5	16.3	-2.0	n.d
10	4.8	16.2	13.7	n.d

^a^calculated from the spectra in Fig 1.

^b^measured in the spectra of Fig 1.

^c^calculated from the spectra obtained in the EPR study of ACE-catalyzed hydrolysis after completion of the reaction (last point in panels A, B, C, D of Fig 4, spectra not shown).

Taken together, the EPR and CD data point to the fact that, even though in aqueous solution the peptides are in equilibrium between different conformations, when labeled at different positions, the peptides display propensities to acquire different conformations. Indeed, when the CD spectra were acquired in the presence of increasing concentrations of TFE, a solvent known to stabilize peptide secondary structure [42], the spectra clearly show that, while the peptides N-terminus displays a propensity to present a less ordered structure, their C-terminal region displays a propensity to form turns (Fig 2). It should be noticed that the EPR and CD data are in remarkable agreement and it can be concluded that the calculated rotational correlation times contain the contribution of both more restricted motion at more internal chain positions and of TOAC-induced bends, both at internal positions and in the peptides C-terminal region. A turn formation at the C-terminal region is also in agreement with ^1^H NMR data [12–13] and molecular dynamics simulations [15] that indicate stabilization of a turn in AngI’s C-terminus.

### CD studies

The Fig 2 shows the CD spectra of AngI and its TOAC-analogues in aqueous solution as a function of increasing TFE concentrations. Of note, AngI (Fig 2A) and biologically active TOAC^0^-AngI and TOAC^1^-AngI (Fig 2B and 2C, respectively) displayed rather similar spectra, typical of an equilibrium between flexible conformers. It should be noticed that, while the pK of the amino group of N-terminally TOAC-labeled peptides is ca. 4.7–4.9 [16–17], pKs found in the literature for this group in AngII range between 6.9 and 7.6 [43–45]. Thus, while the amino groups of TOAC^0^-AngI and TOAC^1^-AngI are fully uncharged at pH 7.2, in the case of the parent compound and the other analogues, an equilibrium probably exists between the protonated and unprotonated forms. Nevertheless, as pointed out by Lintner *et al*. [46], and as suggested by our previous CD titration studies [22], and by the similarity of the CD spectra of AngI, TOAC^0^-AngI, and and TOAC^1^-AngI in aqueous medium (Fig 2A, 2B and 2C, respectively), deprotonation of the terminal amino group seems to affect the peptides conformation to a slight extent.

Concerning the analogues labeled at positions 3, 5, 9, and 10 (Fig 2D, 2E, 2G and 2H, respectively), the spectra are suggestive of the presence of some structure, even in aqueous solution. This is probably due to the TOAC-imposed bend along the peptide backbone. Interestingly, this occurs even in the case of the C-terminal residue (TOAC^10^-AngI). On the other hand, TOAC^8^-AngI gave rise to a spectrum characteristic of an unordered structure in aqueous solution (Fig 2F). This does not seem to corroborate the high τ_C_ value calculated for this analogue’s EPR spectrum. It is conceivable that the features of the CD spectrum are a consequence of the Pro^7^-TOAC^8^ sequence in this peptide.

The CD spectra show that different secondary structures, suggestive of different types of β-turns [47], are stabilized with increasing TFE. In 90% TFE, the spectra of the native peptide (Fig 2A), TOAC^0^-AngI (Fig 2B), and TOAC^1^-AngI (Fig 2C) present similar features, with two minima at 202–205 nm and 214–224 nm, suggesting the presence of type I β-turns. The spectra show that the peptides conformation is essentially unaffected when TOAC is located at the N-terminus. In the case of TOAC^3^-AngI (Fig 2D), TOAC^5^-AngI (Fig 2E), and TOAC^10^-AngI (Fig 2H), positive values of ellipticities are observed at 194–199 nm, and two minima occur between 206–209 nm and 224–234 nm, pointing to the stabilization of type I β-turn spectra. Interestingly, this is also seen for TOAC^10^-AngI. As mentioned above, this result is in full agreement with the turn found for AngI at the C-terminus [12–13, 15]. In contrast, the spectra of TOAC^9^-AngI (Fig 2G) reveal quite distinct features, with a maximum at *ca*. 201 nm and negative ellipticity values spreading from 215 to 240 nm, indicating the formation of a type II β-turn.

As mentioned above, muscle contraction experiments indicated that only AngI analogues labeled at the N-terminus (TOAC^0^-AngI and TOAC^1^-AngI) were able to exert partial activity, in agreement with the known lesser importance of the N-terminal region for this activity [38–39]. In addition, the observation of TOAC^1^-AngI-promoted muscle contraction is in agreement with the work of Cordopatis and Theodoropoulos who showed that Aib^1^-AngII displays high potency in thoracic rabbit aorta strips [48].

Nevertheless, when AngI and its TOAC-labeled analogues were examined with regard to their ability to act as substrates for ACE (Table 1), it was found that, in addition to TOAC^0^- and TOAC^1^-AngI, both TOAC^3^- and TOAC^5^-AngI were also converted into their AngII counterparts, albeit at a slower rate. As mentioned above, taken together, these data show that a much finer control exists for AngII-receptor binding, the initial step of signal transduction, than for AngI binding at the catalytic site of ACE.

### Fluorescence studies

**Fig 3:**
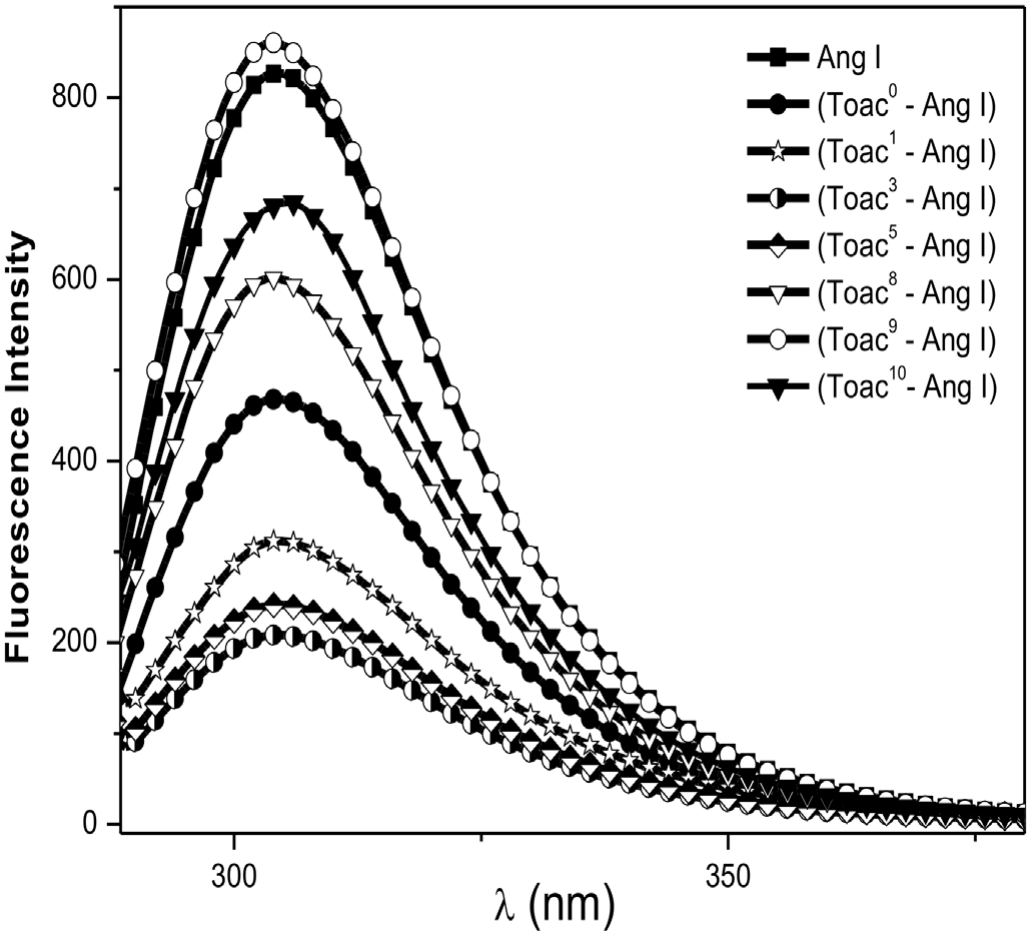
Fluorescence spectra of AngI and TOAC-AngI analogues in 20 mM phosphate buffer, pH 7.

The Fig 3 shows the fluorescence spectra of AngI and its TOAC-labeled analogues. Spectra of the peptides labeled at positions 1, 3, 8, and 9 were previously published [31] and are included for the sake of comparison. The known quenching effect of the nitroxide group [23, 31] upon the fluorescence of the natural AngI Tyr^4^ residue was detected and varied in the order: TOAC^3^-AngI > TOAC^5^-AngI > TOAC^1^-AngI > TOAC^0^-AngI > TOAC^8^-AngI > TOAC^10^-AngI > TOAC^9^-AngI (Table 2). The maximum effect was observed for Tyr^4^ next neighbors, TOAC^3^-AngI and TOAC^5^-AngI; interestingly, residues at the peptide’s N-terminal region exert a much stronger effect than those at the C-terminus, TOAC^9^-AngI having no effect.

These results could be a consequence of the greater flexibility of the N-terminal region, which would allow for a closer average proximity of these residues to Tyr^4^, while the turn at the C-terminal region, by decreasing the peptide chain flexibility, would lead to a lesser degree of quenching. Thus, the fluorescence data are in full agreement with both EPR and CD results.

### Monitoring enzyme kinetics through time-dependent changes of TOAC-labeled peptides EPR spectra

We monitored the enzymatic hydrolysis of TOAC-containing AngI analogues by means of time-dependent changes of their EPR spectra. In this approach, as the peptides are cleaved by ACE at the 8–9 scissile bonds, the time-course of the enzymatic reaction detects the spectral contribution of both compounds: the TOAC-labeled AngI substrate and its enzymatic AngII product, giving rise to two-component spectra. Due to the fact that both peptides differ only by two amino acid residues, their molecular weights are very similar (1,296 Da, native AngI, and 1,046 Da, native AngII), and the EPR spectra of their TOAC counterparts are very similar and essentially indistinguishable.

**Fig 4:**
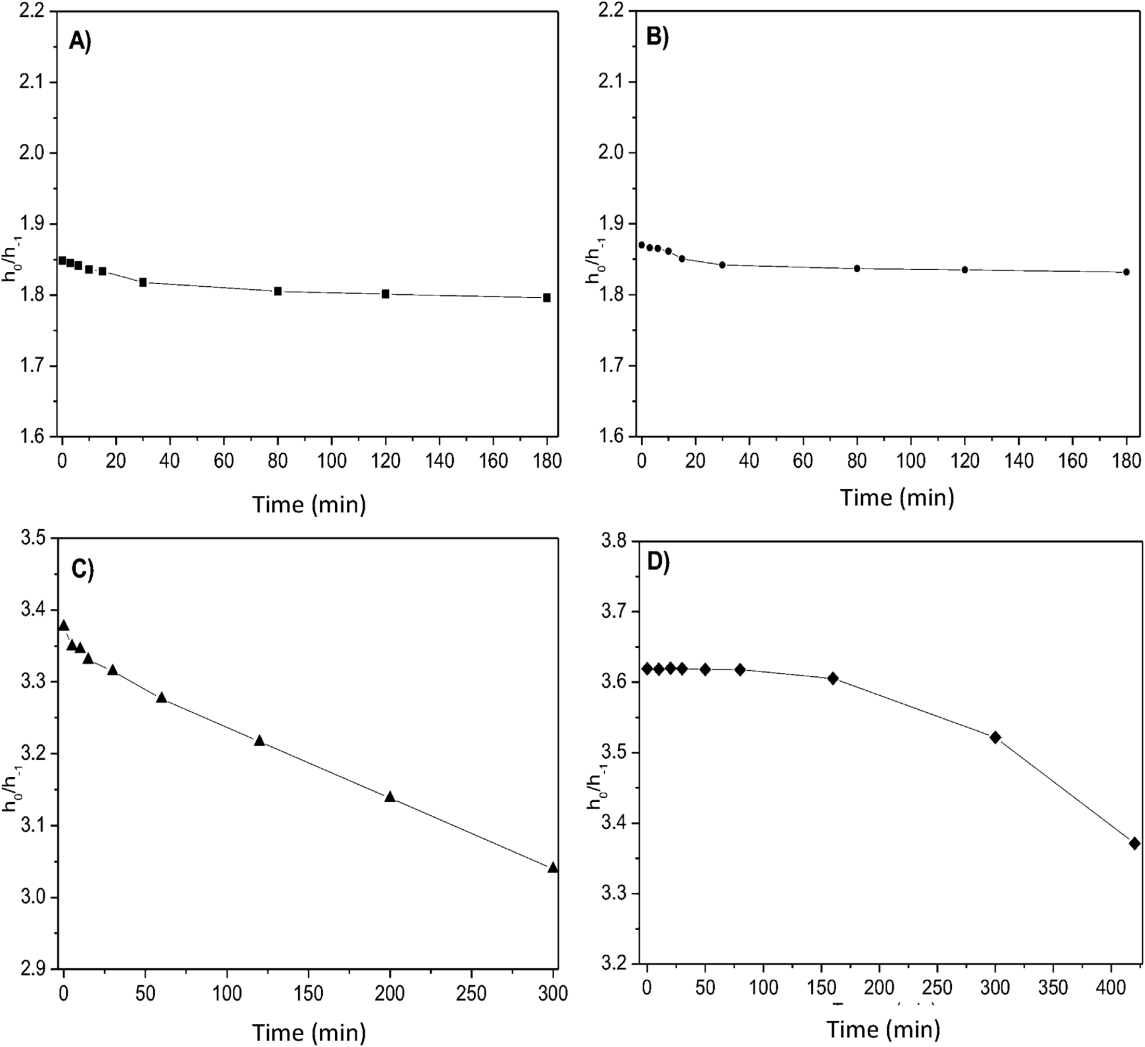
Kinetics of ACE-catalyzed hydrolysis of TOAC^0^-AngI (A), TOAC^1^-AngI (B), TOAC^3^-AngI (C), and TOAC^5^-AngI (D) monitored by the variation of the h_0_/h_-1_ values in the peptides EPR spectra as a function of time.

Still, in spite of the small size difference, it is possible to monitor the kinetics of hydrolysis by building plots of the empirical spectral parameter h_0_/h_-1_ (ratio of heights of the mid-field and high-field lines) as a function of time. This parameter bears a correlation with the rotational correlation time, τ_C_; the higher the correlation time, the higher h_0_/h_-1_ [36]. Since the substrate is a larger molecule, its rotational correlation time, τ_C_, is higher than that of the product (Table 2). Thus, the h_0_/h_-1_ ratio is higher for the substrate spectrum. As the reaction proceeds, the product increasingly contributes to the overall spectrum, and h_0_/h_-1_ progressively decreases (Fig 4).

The reaction was followed until essentially 100% of the substrate was converted into product. The abscissa in Fig 4 evince that the time to reach 100% increases in the order TOAC^0^-AngI = TOAC^1^-AngI < TOAC^3^-AngI < TOAC^5^-AngI, suggesting that, although the two latter peptides are still capable to act as substrates for ACE, very likely their accommodation in the active site is more difficult due to the TOAC-induced turns in the molecules.

## Conclusions

The investigation of peptides comprehending an almost complete TOAC-scan of the AngII precursor—AngI—encompassed both the evaluation of their conformational properties through spectroscopic methods and the examination of their pharmacological properties and ability to act as substrates of the metallodipeptidase ACE in order to establish structure-function relationships for these analogues.

In this context, only analogues attaching TOAC at the N-terminal end (TOAC^0^-AngI and TOAC^1^-AngI) retained partial muscle contractile activity in guinea pig ileum and rat uterus. The lack of activity of derivatives carrying TOAC as residues 3–10 was likely due to the known turn-promoting properties of this cyclic disubstituted glycine. In contrast, the ability to serve as substrates in the ACE-catalyzed hydrolysis was observed for analogues containing TOAC up to residue 5. These findings indicated that the structural requirements for enzymatic hydrolysis are less selective than those regarding ligand-receptor binding and signal transduction.

EPR experiments indicated faster motion for the N-terminal region as opposed to the peptides C-terminal region, supporting the notion that even in aqueous medium the spectra are influenced by the contribution of more folded conformation(s). Moreover, centrally located residues yielded the highest values of rotational correlation times, pointing to the role of the backbone in restricting the motion of these residues.

Representative CD spectra of more flexible conformers were observed mainly for N-terminally labeled analogues. It is noteworthy that the biologically active analogues (TOAC^0^-AngI and TOAC^1^-AngI) presented CD spectra similar to those of native AngI, suggesting a direct relationship between structure and function for these sequences. All other analogues, labeled either at internal positions or at the C-terminal region gave rise to spectra corresponding to more folded structures. The spectra were characteristic of different types of β-turns, pointing to the sequence-dependence of the type of fold originated by TOAC. A rather interesting event stems from the fact that even TOAC^10^-AngI gave rise to a CD spectrum suggestive of a β-turn, in agreement with ^1^H NMR data for AngI.

The intramolecular quenching effect of paramagnetic TOAC upon Tyr^4^ fluorescence was observed for all peptides, except TOAC^9^-AngI. The degree of quenching paralleled the results found by EPR and CD, namely, besides the more effective quenching by the next neighbors, the more flexible N-terminal region was capable of greater quenching than the more rigid C-terminal region.

Finally, we used the time dependence of the EPR spectra to monitor the kinetics of ACE-catalyzed hydrolysis, in spite of the small difference between the spectra of the TOAC-labeled AngI analogues and those of their two-residue shorter products. All analogues containing the spin label inserted up to residue 5 were acceptable as ACE substrates. This approach should be useful in studies of reactions involving TOAC-labeled peptides.
